# Water Adsorption to Leaves of Tall *Cryptomeria japonica* Tree Analyzed by Infrared Spectroscopy under Relative Humidity Control

**DOI:** 10.3390/plants9091107

**Published:** 2020-08-27

**Authors:** Wakana A. Azuma, Satoru Nakashima, Eri Yamakita, Tamihisa Ohta

**Affiliations:** 1 Graduate School of Agricultural Science, Kobe University, Kobe 675-8501, Japan; 2 Graduate School of Science, Osaka University, Osaka 560-0043, Japan or SatoruNakashima.ed@gmail.com (S.N.); eyamakita@ess.sci.osaka-u.ac.jp (E.Y.); 3 Faculty of Environmental and Urban Engineering, Kansai University, Osaka, Suita 564-8680, Japan; 4 Research Institute for Natural Environment, Science and Technology (RINEST), Tarumi-cho 3-6-32 Maison Esaka 1F, Suita, Osaka 564-0062, Japan; 5 Department of Environmental Biology and Chemistry, Graduate School of Science and Engineering, University of Toyama, Toyama 930-8555, Japan; tamihisa@sci.u-toyama.ac.jp

**Keywords:** water molecules, hydrogen bonding, polysaccharides, Cupressaceae, leaf water storage

## Abstract

Leaf water storage is a complex interaction between live tissue properties (anatomy and physiology) and physicochemical properties of biomolecules and water. How leaves adsorb water molecules based on interactions between biomolecules and water, including hydrogen bonding, challenges our understanding of hydraulic acclimation in tall trees where leaves are exposed to more water stress. Here, we used infrared (IR) microspectroscopy with changing relative humidity (RH) on leaves of tall *Cryptomeria japonica* trees. OH band areas correlating with water content were larger for treetop (52 m) than for lower-crown (19 m) leaves, regardless of relative humidity (RH). This high water adsorption in treetop leaves was not explained by polysaccharides such as Ca-bridged pectin, but could be attributed to the greater cross-sectional area of the transfusion tissue. In both treetop and lower-crown leaves, the band areas of long (free water: around 3550 cm^−1^) and short (bound water: around 3200 cm^−1^) hydrogen bonding OH components showed similar increases with increasing RH, while the band area of free water was larger at the treetop leaves regardless of RH. Free water molecules with longer H bonds were considered to be adsorbed loosely to hydrophobic CH surfaces of polysaccharides in the leaf-cross sections.

## 1. Introduction

Within a tree crown, leaves are the primary physiological interface between the plant and the atmosphere and are constantly subject to hydraulic fluctuations. Transpiration through leaves, which is a driving force for water transport from roots to treetop leaves and is important for photosynthesis, changes every moment in response to micrometeorological factors such as solar radiation and vapor pressure deficit [1]. Typically, in response to drought, leaves adjust their stomatal aperture to prevent chronic water stress [2,3] and osmotic capacity to maintain positive turgor pressure and high relative leaf water content [4,5,6].

As trees become tall, however, water transportation is physically difficult due to elongated water transport length and increased gravitational pressure [7,8,9]. Such hydrostatic limitation in tall trees could constrain exploitation of light in upper crown leaves by limiting leaf morphological development, thereby contributing to reduced photosynthetic rates [10,11]. In some tall trees, such as 100-meter tall *Sequoia sempervirens* and *Sequoiadendron giganteum* and 50-meter tall *Cryptomeria japonica*, high leaf water storage partially compensates hydraulic constraint at the treetop [12,13,14,15]. Leaf water storage can function as a hydraulic buffer at the treetop where leaves are exposed to more water stress, particularly in tall trees [13,14,16,17,18]. Regarding whole-tree water use, leaf water storage is important to maintain daily transpiration, especially during drought conditions [19,20,21]. Trees continue to grow taller to get more high solar radiation, but this is a trade-off with water stress. Therefore, the method by which leaves store water in tall trees needs further physicochemical investigation.

With the recent development of spectroscopic techniques, leaf water contents were able to be evaluated by terahertz spectroscopy [22,23]. However, detailed mechanisms of changes in water contents in response to environmental conditions remain unclear. Leaf water storage is a complex interaction between live tissue properties (anatomy and physiology) and the physicochemical properties of the biomolecules or water itself. Water molecules are considered to be present in various hydrogen bond distances, which can be detected as OH stretching absorption frequency shifts to lower wavenumber regions with decreasing intermolecular hydrogen bond distances by infrared (IR) spectroscopy [24]. Bands at around 3550 cm^−1^ and 3200 cm^−1^ can be considered to correspond to long and short hydrogen bonding OH components, called free water and bound water, respectively [25,26,27]. Thus, the hydrogen bonding nature of water molecules associated with biomacromolecules can be studied via IR spectroscopy [26,28,29].

Azuma et al. (2017) [25] conducted a pilot study by IR microspectroscopy on leaf cross-sections of 50-meter tall *C. japonica* trees. OH band areas of IR spectra increased with increased water contents, shown to be larger for leaves at higher heights. These results were consistent with the physiological trend of leaf water storage increasing from the lower crown to the treetop. In addition, the C-O band areas of the IR spectra, including hemicellulose and pectin-like polysaccharide components, were larger in the vascular bundle, transfusion tissue, and epidermis associated with the larger OH band area. Therefore, based on IR spectral analyses, it was inferred that polysaccharides play a key role in water adsorption in leaves of tall *C. japonica*.

In order to explain these results, a working hypothesis was proposed in which water could be adsorbed in Ca-bridged pectin chains in plant cell walls [25]. It is known that plant cell walls include Ca-bridged pectin, which is required for normal growth and development [30,31,32,33] and function to tighten the cell wall structure due to gel formation. This hypothesis was experimentally verified on pectin films with and without Ca by IR microspectroscopy equipped with a relative humidity (RH) control system [27]. Water contents were larger for the pectin film with Ca than without Ca, suggesting that more water molecules were adsorbed by network structures in the Ca cross-linked pectin, with bound water firmly bound to two C=O groups of COOH and free water mainly bound to C=O and C-OH. In order to confirm this Ca-bridged pectin hypothesis in real tall tree leaves, it is necessary to examine Ca contents in the leaves of tall *C. japonica*.

Another recent study regarding the interaction between biomolecules and water showed that in collagen and lecithin films, the band area of free water increased with increasing RH, showing a negative correlation with band areas of aliphatic CH stretching bands [26,34,35]. This interaction between aliphatic CH chains and water molecules was shown to be similar to hydrophobic hydration reported in aliphatic alcohols [36,37,38]. For lecithin, bound water molecules with shorter H bonds are adsorbed to polar phosphate groups at low RH, and further free water molecular layers are added with increasing RH, interacting loosely to aliphatic chains [33]. Because structures of cellulose, hemicellulose, and pectin, which are the main components of plant tissues, have CH, CH_2_, and CH_3_ groups adjacent to polar functional groups, it is possible that these hydrophobic CH groups near polar functional groups of biomolecules can adsorb water molecules with longer H bonds.

**Figure 1:**
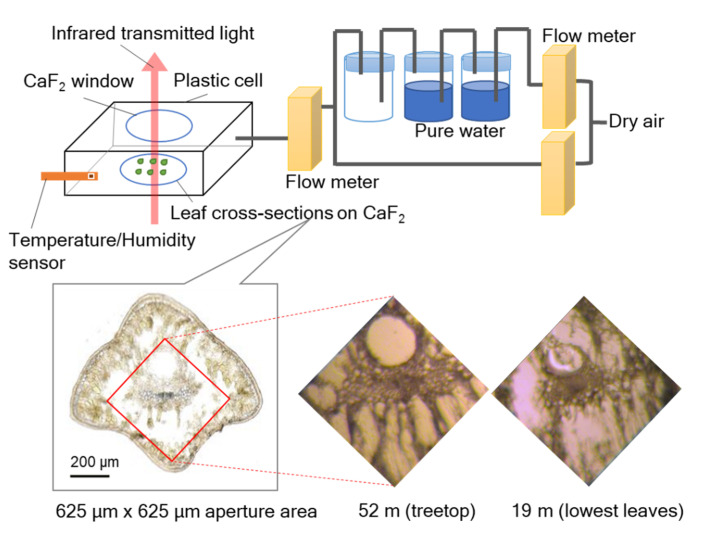
A schematic figure of infrared (IR) microspectroscopy combined with a relative humidity (RH) control system.

In order to examine these hypotheses (i.e., storage in Ca-bridged pectin and interaction with aliphatic CHs of polysaccharides) for water retention in tall tree leaves, we conducted IR microspectroscopy on leaf cross-sections of *C. japonica* from the treetop (52 m) and the lower crown (19 m) using an RH control system (Figure 1). This new system was developed [26,27,34,35] after presentation of the initial results of IR microspectroscopy on leaf cross-sections obtained under equilibrium with a particular room temperature/humidity condition in Azuma et al. (2017) [25]. Changes with RH in band areas of OH (bound and free water), several functional groups, and aliphatic CHs were examined to investigate the water adsorption mechanisms in the leaves of tall trees. We conducted measurements of bulk leaf physiological and anatomical characteristics to coordinate with the results of IR microspectroscopy. In addition, we analyzed Ca contents in treetop and lower-crown leaves to examine the working hypothesis of water adsorption by Ca-bridged pectin.

## 2. Results

### 2.1. Changes with RH in IR Spectra

**Figure 2:**
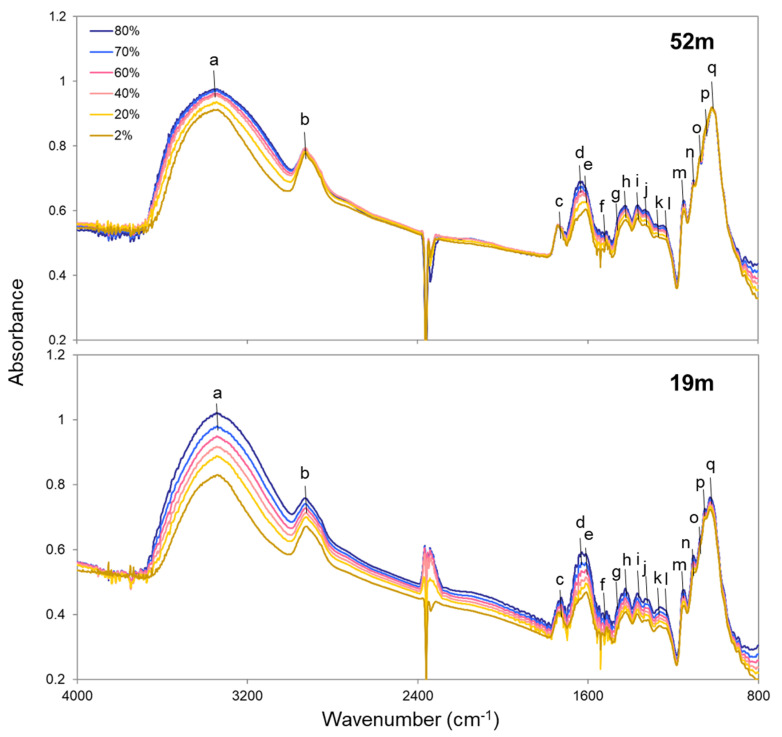
Infrared spectral changes with relative humidity of leaf cross-sections at 52 m and 19 m. The assignments of bands with labels are summarized in Table 1.

Figure 2 shows IR transmission spectra of cross-sections of treetop (52 m) and lower-crown (19 m) leaves with increasing RH (2%, 20%, 40%, 60%, 70%, and 80%). The assignments of IR bands are summarized in Table 1 with corresponding alphabet labels in the IR spectra (Figure 2). It should be noted that bands around 2350 cm^−1^ were due to CO_2_ [39] in the experimental room, which fluctuated mainly due to human respiration (Figure 2).

The peak positions in this study were almost the same as those presented by Azuma et al. (2017) [25], who sampled leaves from the same tree individual and height. With increasing RH, peak shifts were not observed in any peak positions, but the peak heights changed. Therefore, changes with RH in IR band areas were examined as follows.

### 2.2. Changes with RH in IR Band Areas

**Figure 3:**
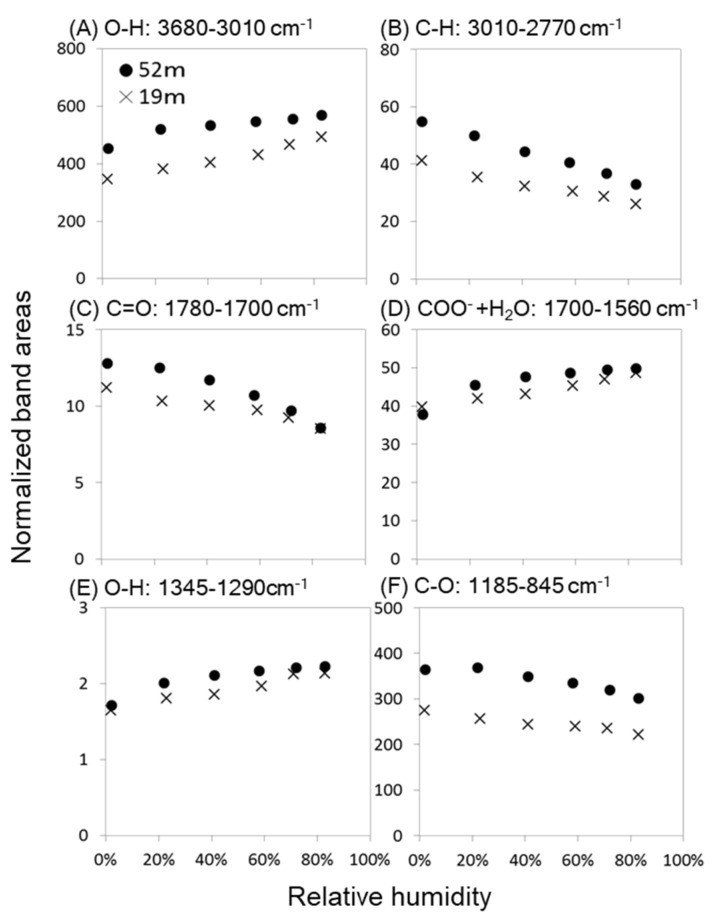
Changes with relative humidity in IR band areas normalized to C=C of lignin (1523–1506 cm^−1^) for cross-sections of treetop (52 m, ●) and lower-crown (19 m, ×) leaves: (**A**) O-H (3680–3010 cm^−1^), (**B**) C-H (3010–2770 cm^−1^), (**C**) C=O (1780–1700 cm^−1^), (**D**) COO^−^ + H_2_O (1700–1560 cm^−1^), (**E**) O-H (1345–1290 cm^−1^), and (**F**) C-O (1185–845 cm^−1^). All relationships showed significant linear relations (*p* < 0.01, Table S1). In all of the relationships, the treetop means were significantly larger than those of the lower-crown leaves in terms of analysis of covariance (*p* < 0.01, Table S3), except for Figure 3D (*p* = 0.04).

All band areas, normalized to that of C=C of lignin (1523–1506 cm^−1^), were larger for treetop (52 m) than for lower-crown (19 m) leaves at all RH conditions (Figure 3 and Table S1). Changes in band areas with increasing RH showed similar trends for both treetop and lower-crown leaves. OH band areas (3680–3010 cm^−1^, Figure 3A; 1345–1290 cm^−1^, Figure 3E) increased with increasing RH. The COO^−^ + H_2_O band (1700–1560 cm^−1^, Figure 3D), including contributions of COO^−^ species (deprotonated COOH) and water molecules (bending), also increased with RH. These results were understood to be due to increasing water adsorption to leaves with increasing RH.

On the other hand, IR band areas of C-H (3010–2770 cm^−1^, Figure 3B), C=O (1780–1700 cm^−1^, Figure 3C), and C-O (1185–845 cm^−1^, Figure 3F) decreased with RH. These IR bands were adjacent to the IR band regions of OH species (see Table 1 and Figure 2). Therefore, some of these IR regions could be lifted due to the influence of increased water molecules with RH, resulting in decreased IR band areas. *R^2^* and *p* values of the regression analyses are summarized in Table S2.

### 2.3. Changes with RH in OH Band Components

**Figure 4:**
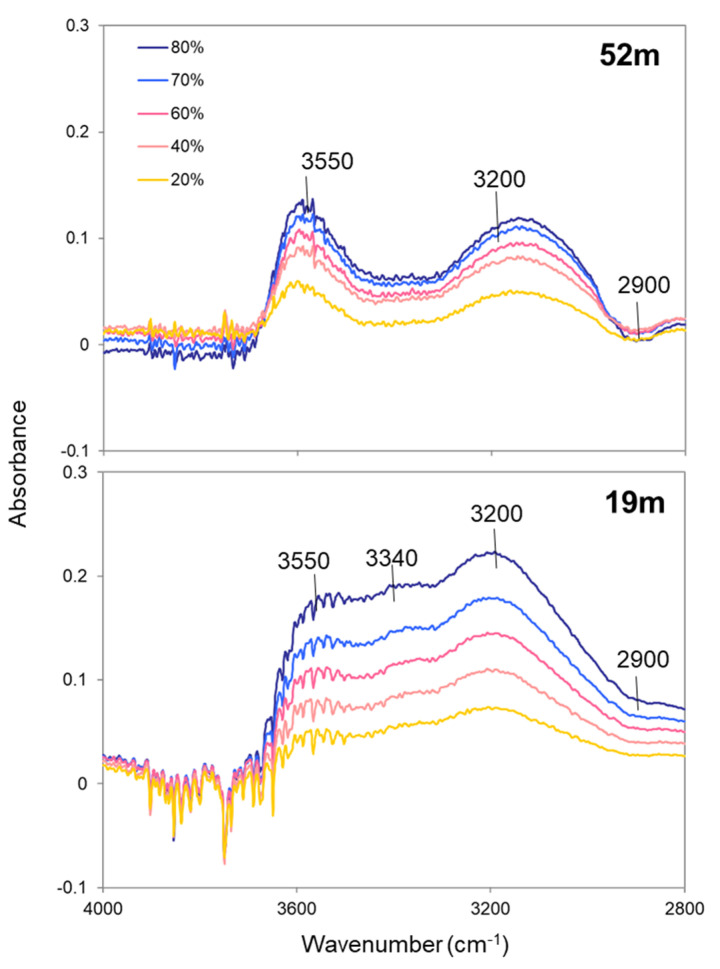
Difference spectra from the driest cross-section (RH = 2%) at different RH levels of leaf cross-sections at 52 m and 19 m.

To examine the detailed changes in the OH band components with increasing RH, difference spectra from the driest leaf cross-section were examined (Figure 4). In the OH stretching region (3680–3010 cm^−1^) of the difference spectra for the treetop (52 m) and lower-crown (19 m) leaves, positive bands were observed at 3550 and 3200 cm^−1^ (Figure 4). As explained in the introduction, the bands at 3550 and 3200 cm^−1^ can be considered to correspond to long and short hydrogen bonding OH components, called free water and bound water, respectively [25,26,27].

**Figure 5:**
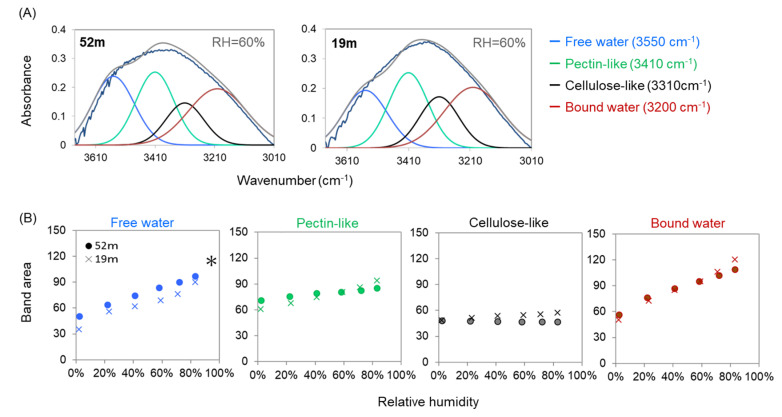
(**A**) Representative curve fitting results by four Gaussian OH components with initial band positions at 3550 cm^−1^ (water molecules with longer H bonds), 3410 cm^−1^ (-COOH groups in pectin-like species), 3310 cm^−1^ (OH groups in cellulose-like species) and 3200 cm^−1^ (water molecules with shorter H bonds) in leaf cross-sections at 52 m and 19 m under RH = 60%. (**B**) Changes with relative humidity in IR band areas of four OH band components for leaf cross-sections at the treetop (52 m, ●) and the lower crown (19 m, ×). All of the relationships showed significant linear relations (*p* < 0.01, Table S1). Asterisks indicate the means of the treetop were significantly larger than those of the lower crown in terms of analysis of covariance (*p* < 0.01, Table S3).

In order to detect changes in OH band components with RH, the OH band positions in free water (3550 cm^−1^), bound water (3200 cm^−1^), OH groups in cellulose-like species (3310 cm^−1^), and -COOH groups in pectin-like species (3410 cm^−1^) were used as fixed positions to fit the OH bands of leaves by four Gaussian bands. Representative fitting results of treetop (52 m) and lower-crown (19 m) leaves at RH = 60% are shown in Figure 5A. All four OH band components changed with increasing RH, with marked increases in free water and bound water (Figure 5B and Table S1). The band areas of free water were consistently larger in the treetop than in the lower crown, while those corresponding to the bound water were mostly similar between these two regions; however, the bound water increased more at higher RH levels in the lower-crown region (Figure 5B and Table S3).

### 2.4. Changes with RH in CH Band Area

**Figure 6:**
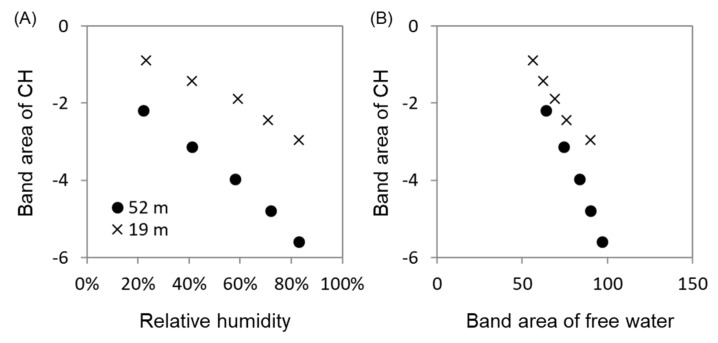
Relationship between IR band areas of C-H (3010–2770 cm^−1^) in difference spectra and (**A**) relative humidity and (**B**) IR band area of free water (water molecules with longer H bonds) for leaf cross-sections at the treetop (52 m, ●) and the lower crown (19 m, ×). All relationships showed clear linear correlations (*p* < 0.01, Table S2). In both (**A**) and (**B**) relationships, the means of the treetop were significantly smaller than those of the lower crown in terms of analysis of covariance (*p* < 0.01, Table S3).

We analyzed the changes with RH in the CH band area (the band at 2900 cm^−1^ in Figure 3) by using the difference spectra, excluding the influence of the band tails of adjacent water. CH band areas of the treetop and lower-crown leaves decreased with RH (Figure 6A) and were negatively correlated with the band area of the free water (Figure 6B).

### 2.5. Bulk Leaf Water Storage, Inorganic Element Concentrations, and Tissue Compositions of Leaves

Leaf capacitance (*C_leaf_*) and succulence (*S_leaf_*) were larger in the treetop (52 m) than in the lower-crown (19 m) leaves (*p* < 0.01, Student’s *t*-test). *C_leaf_* and *S_leaf_* values of the treetop leaves were almost twice those of the lower-crown leaves (Table 2).

Concentrations of inorganic elements are larger in the following order in both the treetop and in the lower-crown leaves: Ca > K > Mg > P > Fe. It should be noted that Ca and Mg concentrations were generally smaller in the treetop than in the lower crown (Table 2).

Tissue compositions within IR measurement areas of the cross-sections were different between the treetop and lower-crown leaves. The cross-sectional area percentages of vascular bundles and transfusion tissue within the IR measurement areas of the cross-sections were larger in the treetop than in the lower-crown leaves. On the other hand, that of mesophyll area was smaller in treetop than in lower-crown leaves (Table 2).

## 3. Discussion

In this study, IR microspectroscopy with changing relative humidity (RH) was conducted on leaf cross-sections of *C. japonica* from treetop (52 m) and lower-crown (19 m) leaves to examine their water adsorption behaviors in the tall tree. Both OH band areas of the treetop and lower-crown leaves increased with increasing RH, showing that water molecules were adsorbed on the leaf cross-sections.

The first key result was that OH band areas were always larger for the treetop than the lower-crown leaves regardless of RH (Figure 3). This was consistent with high bulk leaf water storage for the treetop leaves in tall *C. japonica* reported here (Table 2) and in previous studies using physiological conditions and IR measurements under ambient equilibrium conditions [13,25]. In the leaves of tall *C. japonica*, the vascular bundle functions as a water transportation pathway and transfusion tissue functions as a water-storing agent and a hydraulic buffer, preventing excessive decrease in xylem water potentials [13]. Based on the IR spectral analyses, OH band areas were larger in the vascular bundle and transfusion tissue within leaf cross-sections [25]. Comparing the cross-sectional area percentages of these two tissues within IR measurement areas of the leaf cross-sections of the treetop and the lower crown in this study, those of the vascular bundle were similar, while those of the transfusion tissue were more than twice as large in the treetop than in the lower crown (Table 2), indicating that the surface area of transfusion tissue was larger in the treetop than in the lower crown. Therefore, the larger surface area of transfusion tissue in the treetop leaves can adsorb more water molecules, which may contribute to the high water adsorption capacity of the treetop leaves compared to lower-crown leaves in tall *C. japonica*.

The second key result was the difference in water components adsorbed on the leaves with increasing RH. In both the treetop and the lower-crown leaves, the band areas of free water (the band at 3550 cm^−1^) and bound water (the band at 3200 cm^−1^) showed similar increasing trends with increasing RH, while the band area of free water was larger in the treetop than in the lower crown (Figure 5), indicating that treetop leaves can hold more free water than lower-crown leaves.

In this study, the CH band areas (the band at 2900 cm^−1^) of the treetop and lower-crown leaves decreased with RH (Figure 6A) and were negatively correlated with the band area of free water (Figure 6B). These trends were similar to previous reports of interactions between water molecules and biomolecules, such as collagen and lecithin [26,34,35]. Because the structures of the main components of plant tissues, such as cellulose, hemicellulose, and pectin, have CH, CH_2_, and CH_3_ groups adjacent to polar functional groups, these correlations imply that free water with longer hydrogen bonds could be loosely bound to hydrophobic CH surfaces of polysaccharides in the leaf cross-sections of *C. japonica*. Since the cross-sectional area percentages of transfusion tissue are much larger in the treetop leaves than in the lower-crown leaves (Table 2), free water molecules can be adsorbed loosely onto surface areas of transfusion tissue.

On the other hand, the OH component around 3200 cm^−1^ is considered to be bound water with short H bonds, contained in grain boundaries of minerals and in artificially-made nanopores [56,57,58] and often called “ice-like water” due to its similarity to water ice [59]. In plants, the principal component of the cell wall is cellulose, which forms nanoscale structures called microfibrils. These cellulose microfibrils are embedded in a crosslinked matrix of polysaccharides, such as hemicelluloses and pectin [60]. Water molecules with short H bonds may be adsorbed into these cell wall matrix boundaries.

Based on the IR spectral analyses, a previous study suggested a working hypothesis of water molecules being bound to Ca-bridged pectin molecules by hydrogen bonding to C-O-C and C-OH [25]. This hypothesis was verified using pectin films with and without Ca by the same method used in this study, i.e., the IR method with RH control, showing that water contents were larger for the pectin film with Ca than without Ca [27]. Therefore, water molecules were previously hypothesized to be mainly adsorbed by the Ca-bridged pectin in tall tree leaves [25]. However, the Ca concentration was found to be lower in the treetop leaves than in the lower-crown leaves of tall *C. japonica* (Table 2), suggesting that there is less Ca-bridged pectin in treetop leaves than lower-crown leaves. Therefore, Ca-bridged pectin may not the primary factor of the high-water capacity for treetop leaves in tall *C. japonica*. In addition, the peak heights of hemicellulose and pectin in the C-O band region were not different between treetop and lower-crown leaves (Figure 2), suggesting that they have similar polysaccharide compositions and indicating that these molecules might not affect the difference in water adsorption between the treetop and the lower crown.

## 4. Conclusions

In this study, at least two species of water components with long and short hydrogen bonds (so-called free water and bound water, respectively) were adsorbed under relative humidity changes in both treetop and lower-crown leaves. Regardless of RH, free water was adsorbed more on the treetop leaves than in the lower-crown leaves, possibly because of the greater cross-sectional area of the transfusion tissue. Contrary to the previous hypothesis, Ca-bridged pectin may not the primary factor of high-water capacity for treetop leaves in tall *C. japonica*. The present results suggest that hydrophobic CH groups near the polar functional groups of polysaccharides can adsorb water molecules with longer H bonds. Therefore, free water molecules are considered to be adsorbed loosely on large surface areas of transfusion tissue, while bound water may be held between nanoscopic boundaries of cell wall polysaccharides in leaf cross-sections of tall *C. japonica*.

Although further studies on the interactions between biomacromolecules and water molecules through the nature of hydrogen bonding are still needed for deeper understanding [28,29], IR microspectroscopy of leaves with an RH control system would be a new approach to help further discuss tree physiological functions with physicochemical interactions of water and biomolecules.

## 5. Materials and Methods

### 5.1. Preparation of Samples

Field sampling was conducted at Nibuna-Mizusawa Forest Reserve, Tashirozawa National Forest in Akita Prefecture, Japan (40.08° N, 140.25° E, 200 m altitude from sea level (ASL)) in June 2018. We accessed a single crown of *Cryptomeria japonica* (250 years old, height of 52 m, and diameter at breast height of 114 cm) using a single-rope climbing technique and collected two or three small branches (30–50 cm long) from the outer crown from just below the treetop (52 m) and the lowest living branches (19 m). The tree and sampling heights were the same as those used in Azuma et al. (2017) [25]. Sample branches were immediately recut under water, sealed in black plastic bags, and fully rehydrated in the laboratory overnight for physiological measurement. The rest of the branches were stored in a refrigerator for three days for IR microspectroscopy.

For IR microspectroscopy, because maturation of current-year leaves was not complete, second-year leaves were sectioned at a thickness of 24 µm at the midpoint between the leaf tip and its stem attachment using a sliding microtome equipped with frozen mounting (REM-710, Yamato Kohki Industrial Co., Ltd., Saitama, Japan). These leaf cross-sections were mounted on an IR transparent CaF_2_ crystal.

### 5.2. Bulk Leaf Water Storage

Three shoots comprising second- and current-year internodes were removed from the sampled branches at each height for measurement of bulk leaf water storage. Leaf capacitance (*C_leaf_*, mol·m^−2^ MPa^−1^ [25,61]) was obtained from a pressure–volume curve using the bench-drying approach [62,63] with a pressure chamber (Model 1000, PMS Instruments, Corvallis, OR, USA). All sample shoots were photographed for measurement of the leaf surface area. Saturated leaf water contents (WW, g) were calculated by subtracting the dry weights (after drying to constant weights at 65 °C for 48 h) from fresh leaf weights at water saturation. WWs were normalized by the leaf surface area to obtain saturated leaf water contents per unit area or succulence (*S_leaf_*, g·H_2_O·m^−2^; [25,64]).

### 5.3. Inorganic Elements of Leaves

Three shoots used for the above measurements of bulk leaf water storage were dried at 65 °C for 48 h in an oven and then ground with a ball mill. These powder leaf samples were decomposed with 70% HNO_3_ using a microwave oven (ETHOS One, Milestone, Sorisole, Italy). The obtained extracts were evaporated on a hot plate (80 °C) in a clean room, and the evaporation residues were dissolved in 1% HNO_3_. Then, inorganic element concentrations per unit dry mass in leaves (mg·g^−1^) were measured using inductively coupled, plasma atomic emission spectrometry (iCAP 6200, Thermo Fisher Scientific, Cambridge, UK). The inorganic elements in the sample solutions were quantified using an internal standard solution (yttrium), which was premixed with sample extracts.

### 5.4. IR Microspectroscopy with a Relative Humidity Control System

To examine water adsorption to leaf cross-sections under changes in water vapor pressure, transmission IR spectra were measured using a Fourier-transform infrared (FT-IR) microspectrometer (FTIR-680 + IRT30, Jasco, Tokyo, Japan) with a relative humidity (RH) control system, as described above (Figure 1). First, a CaF_2_ crystal, on which leaf cross-sections were mounted, was put into a plastic cell (36 × 36 × 14 mm) with a CaF_2_ window at room temperature (around 25 °C). Then, the RH in the cell was controlled by changing the flow rate of dry air provided by a dehumidifier (AM-12, Jasco, Tokyo, Japan) and air through two bottles of pure water with digital flowmeters (D8500, Kofloc, Kyoto, Japan). Fluctuations in temperature and humidity in the cell were monitored every second by a small sensor (SHT35, Sensirion, Stäfa, Switzerland) equipped with a data logger (SCHM-1, Syscom, Tokyo, Japan). During the IR measurement for leaf cross-sections, RH was increased every ca. 30 min stepwise from ca. 2%, 20%, 40%, 60%, 70%, to 80%, and the temperature was constant at ca. 29 °C (Figure S1). Under such RH conditions, sample transmission spectra of 625 µm × 625 µm aperture size (Figure 1) were measured through the CaF_2_ window every 1 min, with 64 scans at 4 cm^−1^ resolution in the 4000–800 cm^−1^ region.

Microscopic images of the leaf cross-sections were taken under an optical microscope, then component area ratios of each tissue (mesophyll, vascular bundle, and transfusion tissue (including sheath cells)) were measured using Image J.

### 5.5. IR Band Area Changes with RH

To examine IR band changes due to changing RH, the following IR band areas were measured with baselines set for each band region by Spectra Manager software (Jasco, Tokyo, Japan): OH stretching vibrations in the 3680–3010 cm^−1^ region, stretching of aliphatic CH groups in the 3010–2770 cm^−1^ region, C=O stretching vibrations of carboxyl groups in the 1780–1700 cm^−1^ region, bending vibrations of COO^−^ and H_2_O molecules in the 1700–1560 cm^−1^ region, C=C of lignin in the 1523–1506 cm^−1^ region, C-H bending vibrations in the 1475–1440 cm^−1^ region and 1390–1345 cm^−1^ region, COO^−^ asymmetric and symmetric stretching vibrations in the 1440–1390 cm^−1^ region, OH bending in the 1345–1290 cm^−1^ region, C-C and C-O stretching of carbohydrates and lignin and OH symmetric bending in the 1290–1185 cm^−1^ region, and C-O stretching in the 1185–845 cm^−1^ region (Figure 2, Figure S2). The assignments of these bands are summarized in Table 1. Since the IR band area of C=C of lignin in the 1523–1506 cm^−1^ region was the most stable component at any RH, it was used for normalization of the IR band areas. Changes in the normalized band areas according to RH are shown in Figure 3.

### 5.6. Changes in OH Band Components with RH

To detect changes in OH band components with increasing RH, IR spectra at RH of 20%, 40%, 60%, 70%, and 80% were subtracted by the driest leaf cross-section spectrum at 2% RH. In the difference spectra of the cross-sections of treetop (52 m) and lower-crown (19 m) leaves, positive bands at around 3550 cm^−1^ and 3200 cm^−1^ were observed in the OH stretching region (Figure 4). According to Azuma et al. (2017) [25], the OH band positions observed in the difference spectra of leaves (3550 and 3200 cm^−1^) were considered to be representative OH band components of water molecules, which are different from OH groups in cellulose-like species (3310 cm^−1^) and -COOH groups in pectin-like species (3410 cm^−1^). It should be noted that the difference spectra for the leaves at 19 m in this study showed a band around 3340 cm^−1^ (Figure 4), but this may have only been an apparent band due to contributions from cellulose and pectin-like OHs. Therefore, these four band positions (3550, 3410, 3310, and 3200 cm^−1^) were used as fixed positions to fit the OH bands of leaves by four Gaussian bands. The band widths of long and short hydrogen bond components (3550 and 3200 cm^−1^) were not fixed because they are considered to vary with RH, while the band widths of cellulose-like and pectin-like species (3410 and 3310 cm^−1^) were fixed at 150 cm^−1^. None of the band heights of the OH band components were fixed.

### 5.7. Changes in CH Band Areas with RH

We analyzed changes with RH in the CH band area (the band at 2900 cm^−1^ in Figure 3) using the difference spectra, excluding the influence of the band tails of adjacent water. IR band areas were measured with baselines set for each band region by Spectra Manager software (Jasco, Tokyo, Japan).

### 5.8. Data Analyses

Bulk leaf water storage and inorganic element concentrations of leaves were compared between treetop (52 m) and lower-crown (19 m) leaves by Student’s *t*-test (Table 2). Changes with RH in the normalized band areas and the band areas of OH band components for cross-sections of the treetop (52 m) and lower-crown (19 m) leaves were analyzed by linear regression analyses (Tables S1 and S2). The effect of the leaf position (treetop vs. lower crown) on changes with RH in IR band areas were analyzed using ANCOVA (analysis of covariance), with the leaf position being the main effect and RH representing the covariates (Table S3). Because the number of studied leaves was small, we applied conservative criteria for statistical significance (*p* < 0.01) in our statistical analyses. We used JMP14.2 software (SAS Institute, Cary, NC, USA) for all analyses.

## Figures and Tables

**Table 1:** Assignments of IR bands for the leaf cross-sections at 52 m and 19 m. The labels correspond to those for the bands shown in Figure 2. Abbreviations: ν_as_, asymmetric stretch; ν_s_, symmetric stretch; δ_as_, asymmetric deformation (bend); δ_s_, symmetric deformation (bend). Band assignments are taken from the listed references.

Labels	Peak Position (cm^−1^)	Assignment	Reference
a	3340	ν(O-H)	[40,41]
b	2920	ν(C-H)	[42,43]
c	1740	ν(C=O) (COOH)	[44,45]
d	1640	δ(H-O-H) of water molecule	[46,47]
e	1610	ν_as_(COO^−^)	[48,49]
f	1515	ν(C=C)	[50]
g	1450	δ_as_(CH_3_)	[50]
h	1415	ν_s_(COO^−^)	[45,48]
i	1370	δ_as_(CH_3_)	[50]
j	1315	δ(O-H)	[40,41]
k	1263	ν(C-O-C)	[51,52]
l	1229	δ(O-H)	[51]
m	1150	ν_as_(C-O-C)	[40,41,53]
n	1105	ν_s_(COOH)	[49]
o	1075	ν(C-O-C), δ(O-H)	[40,53]
p	1050	ν(C-O)	[40,41]
q	1020	ν(C-O)	[40,41,53]
-	700	δ(OH)	[54,55]

**Table 2:** Bulk leaf water storage (leaf capacitance, *C_leaf_*, and succulence, *S_leaf_*: Mean and (SD) of three shoots), inorganic element concentrations per unit dry mass in leaves (mean and (SD) of three shoots), and area percentages of tissues within IR measurement areas of the leaf cross-sections at 52 m and 19 m. Significant differences between the means of 52 m and 19 m are denoted by different lowercase letters (a, b) (Student’s *t*-test, *p* < 0.01).

		52 m	19 m
Bulk leaf water storage	*C_leaf_* (mol·m^−2^·MPa^−1^)	1.4 (0.06) a	0.9 (0.12) b
	*S_leaf_* (g·H_2_O·m^−2^)	226 (6.66) a	125 (4.92) b
Inorganic element concentrations	Ca (mg·g^−1^)	12.37 (0.74) a	19.39 (1.36) b
	Mg (mg·g^−1^)	2.07 (0.09) a	3.31 (0.15) b
	K (mg·g^−1^)	6.01 (0.98) a	5.71 (0.26) a
	Na (mg·g^−1^)	0.11 (0.03) a	0.34 (0.24) a
	P (mg·g^−1^)	1.71 (0.19) a	1.96 (0.15) a
	Fe (mg·g^−1^)	0.07 (0.02) a	0.09 (0.01) a
Area percentages of tissues in leaf cross-sections	vascular bundle (%)	3.2	2.3
	transfusion tissue (%)	15.6	5.8
	mesophyll (%)	70.7	86.6

## Supplementary Materials

The following are available online at https://www.mdpi.com/2223-7747/9/9/1107/s1: Figure S1: Relative humidity changes and constant temperatures in the plastic cell during IR measurement for leaf cross-sections at 52 m and 19 m; Figure S2: Changes with relative humidity in raw IR band areas for leaf cross-sections at 52 m and 19 m; Table S1: Statistical relationships between normalized IR band areas and relative humidity using the shown regression analyses in Figure 3 and Figure 5; Table S2: Statistical relationships using the regression analyses between the CH band areas (2770–3010 cm^−1^) of difference spectra and relative humidity, shown in Figure 6A, and the band area of free water (around 3550 cm^−1^), shown in Figure 6B; Table S3: Results of ANCOVA for IR band areas with leaf position (treetop at 52 m vs. lower crown at 19 m) being the main effect and relative humidity or band area of free water representing the covariates.
