# Supplementary material for: Water Adsorption to Leaves of Tall Cryptomeria japonica Tree Analyzed by Infrared Spectroscopy under Relative Humidity Control
Source: Plants (Basel). 2020 Aug 27;9(9):1107. doi: 10.3390/plants9091107 (PMC7569789; doi:10.3390/plants9091107)

Table S1. Results of ANCOVA for IR band areas with leaf positions (treetop at 52 m vs lower-crown at 19 m) as the main effect and relative humidity or band area of free water as covariates.

Response (IR band area)	effect	DF	SSE	F	P
Fig. 3A; O-H (3680-3010 cm^{-1})	leaf position	1	35988.59	205.17	<0.0001
	relative humidity	1	21237.03	121.07	<0.0001
	interaction	1	583.94	3.33	0.106
	error	8	1403.28		
	total	11	58994.40		
Fig. 3B; C-H (3010-2770 cm^{-1})	leaf position	1	349.63	590.91	<0.0001
	relative humidity	1	456.59	771.68	<0.0001
	interaction	1	20.29	34.30	0.0004
	error	8	4.73		
	total	11	835.19		
Fig. 3C; C=O (1780-1700 cm^{-1})	leaf position	1	3.84	27.91	0.0007
	relative humidity	1	15.54	112.83	<0.0001
	interaction	1	1.18	8.59	0.0190
	error	8	1.10		
	total	11	21.78		
Fig. 3D; COO– + H2O (1700-1560 cm^{-1})	leaf position	1	14.60	5.70	0.044
	relative humidity	1	135.64	52.99	<0.0001
	interaction	1	1.68	0.66	0.441
	error	8	20.48		
	total	11	172.39		

Fig. 3E; O-H (1345-1290 cm^{-1})	leaf position	1	0.068	17.70	0.003
	relative humidity	1	0.334	86.78	<0.0001
	interaction	1	0.0002	0.0508	0.827
	error	8	0.031		
	total	11	0.432		
Fig. 3F; C–O (1185-845 cm^{-1})	leaf position	1	26527.52	570.69	<0.0001
	relative humidity	1	4555.69	98.01	<0.0001
	interaction	1	110.29	2.37	0.162
	error	8	371.86		
	total	11	31642.64		
Fig. 5; Water molecule with long H bonds (around 3550 cm^{-1})	leaf position	1	3169.92	331.46	0.0002
	relative humidity	1	425.24	44.46	<0.0001
	interaction	1	3.04	0.318	0.588
	error	8	76.51		
	total	11	3666.47		
Fig. 5B; –COOH groups in pectin-like species (around 3410 cm^{-1})	leaf position	1	737.75	453.28	0.0475
	relative humidity	1	8.91	5.47	<0.0001
	interaction	1	128.45	78.92	<0.0001
	error	8	13.02		
	total	11	882.85		
Fig. 5B; OH groups in cellulose-like species (around 3310 cm^{-1})	leaf position	1	16.48	120.58	<0.0001
	relative humidity	1	122.32	894.83	<0.0001
	interaction	1	35.79	261.82	<0.0001

	error	8	1.09		
	total	11	175.58		
Fig. 5B; Water molecule with short H bonds (around 3200 cm^{-1})	leaf position	1	4752.37	520.95	0.759
	relative humidity	1	0.920	0.101	<0.0001
	interaction	1	89.41	9.80	0.014
	error	8	72.98		
	total	11	4906.16		
Fig. 6A; CH (2770-3010 cm^{-1})	leaf position	1	10.23	739.94	<0.0001
	relative humidity	1	9.12	659.63	<0.0001
	interaction	1	0.496	35.86	0.001
	error	6	0.083		
	total	9	19.93		
Fig. 6B; CH (2770-3010 cm^{-1})	leaf position	1	2.50	112.67	<0.0001
	band area of free water	1	9.11	411.33	<0.0001
	interaction	1	0.563	25.41	0.002
	error	6	0.133		
	total	9	19.93		

Table S2. Statistical relationships between the normalized IR band areas and relative humidity using the regression analyses in Figs. 3 and 5.

Corresponding Fig.	Band region (cm^{-1})	52m		19m	
		R^2	*p*-value	R^2	*p*-value
Fig. 3A	3680 - 3010	0.84	<0.01	0.97	<0.001
Fig. 3B	3010 - 2770	0.99	<0.001	0.97	<0.001
Fig. 3C	1780 - 1700	0.92	<0.01	0.94	<0.001
Fig. 3D	1700 - 1560	0.76	<0.01	0.97	<0.001
Fig. 3E	1345 - 1290	0.83	<0.01	0.96	<0.001
Fig. 3F	1185 - 845	0.89	<0.01	0.95	<0.001
Fig. 5B	Water molecule with long H bonds (around 3550)	0.99	<0.001	0.95	<0.001
Fig. 5B	−COOH groups in pectin-like species (around 3410)	0.97	<0.001	0.98	<0.001
Fig. 5B	OH groups in cellulose-like species (around 3310)	0.92	<0.01	0.98	<0.001
Fig. 5B	Water molecule with short H bonds (around 3200)	0.97	<0.001	0.99	<0.001

Table S3. Statistical relationships using the regression analyses between CH band area (2770-3010 cm^{-1}) of difference spectra and relative humidity in Fig. 6A and band area of free water (around 3550 cm^{-1}) in Fig. 6B.

Corresponding	52m		19m	
Fig.	R^2	*p*-value	R^2	*p*-value
Fig. 6A	0.99	<0.001	0.98	<0.001
Fig. 6B	0.99	<0.001	0.96	0.002

Fig. S1 Relative humidity changes and constant temperatures in the plastic cell during the IR measurement for leaf cross-sections at 52 m and 19 m.

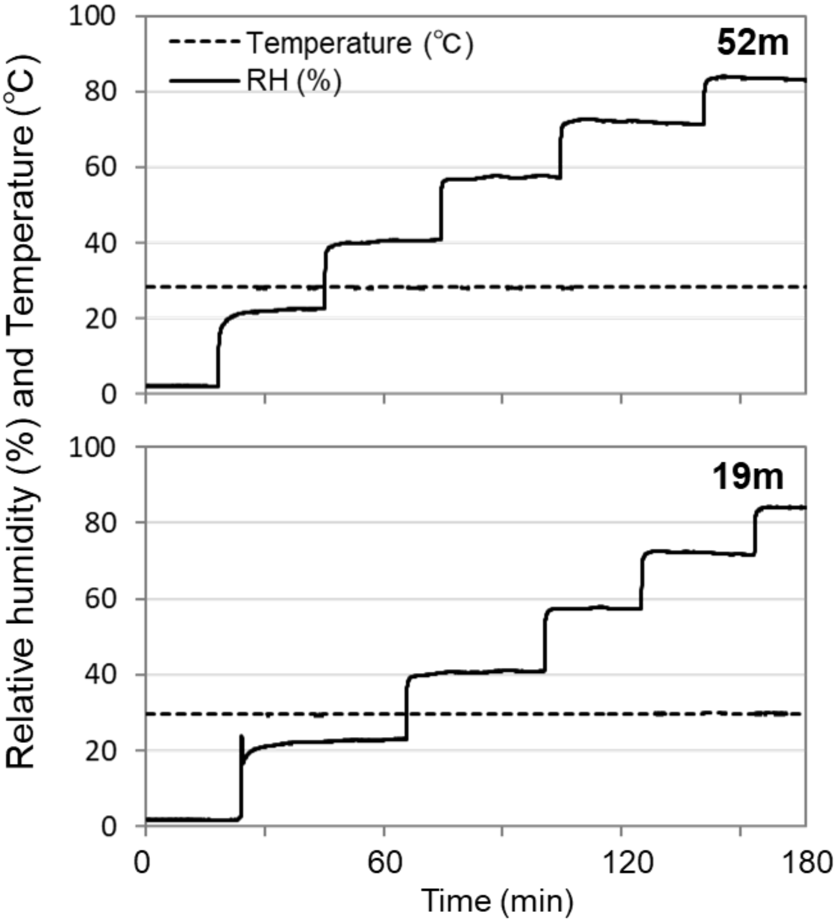

Fig. S2 Changes with relative humidity in raw IR band areas of (A) O-H (3680-3010 cm^{-1}), (B) C-H (3010-2770 cm^{-1}), (C) C=O (1780-1700 cm^{-1}), (D) COO^{-} + H_2O (1700-1560 cm^{-1}), (E) C=C (1523-1506 cm^{-1}), (F) CH (1475-1440 cm^{-1}), (G) COO^{-} (1440-1390 cm^{-1}), (H) CH (1390-345 cm^{-1}), (I) O-H (1345-1290 cm^{-1}), (J) C-O-C + H_2O (1290-1185 cm^{-1}), and (K) C–O (1185-845 cm^{-1}) for leaf cross-sections at 52 m (●) and 19 m (×).

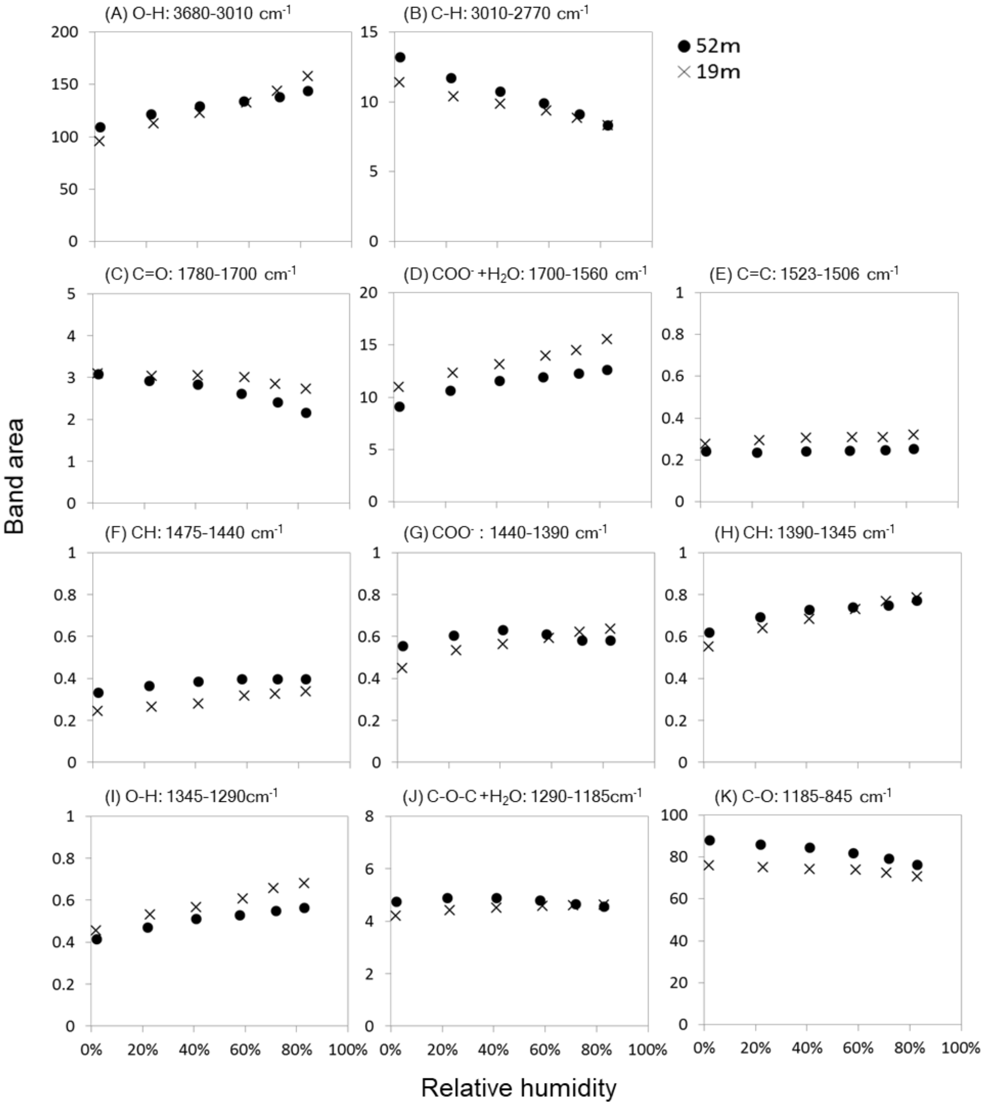

Supplement: Supplementary file 1 [file plants-09-01107-s001.pdf]
